# Autophagy, Warburg, and Warburg Reverse Effects in Human Cancer

**DOI:** 10.1155/2014/926729

**Published:** 2014-08-12

**Authors:** Claudio D. Gonzalez, Silvia Alvarez, Alejandro Ropolo, Carla Rosenzvit, Maria F. Gonzalez Bagnes, Maria I. Vaccaro

**Affiliations:** ^1^Institute of Biochemistry and Molecular Medicine, National Council for Scientific and Technological Research, School of Pharmacy and Biochemistry, University of Buenos Aires, Junin 956 p5, 1113 Buenos Aires, Argentina; ^2^Department of Pharmacology, CEMIC University Institute, 1113 Buenos Aires, Argentina

## Abstract

Autophagy is a highly regulated-cell pathway for degrading long-lived proteins as well as for clearing cytoplasmic organelles. Autophagy is a key contributor to cellular homeostasis and metabolism. Warburg hypothesized that cancer growth is frequently associated with a deviation of a set of energy generation mechanisms to a nonoxidative breakdown of glucose. This cellular phenomenon seems to rely on a respiratory impairment, linked to mitochondrial dysfunction. This mitochondrial dysfunction results in a switch to anaerobic glycolysis. It has been recently suggested that epithelial cancer cells may induce the Warburg effect in neighboring stromal fibroblasts in which autophagy was activated. These series of observations drove to the proposal of a putative reverse Warburg effect of pathophysiological relevance for, at least, some tumor phenotypes. In this review we introduce the autophagy process and its regulation and its selective pathways and role in cancer cell metabolism. We define and describe the Warburg effect and the newly suggested “reverse” hypothesis. We also discuss the potential value of modulating autophagy with several pharmacological agents able to modify the Warburg effect. The association of the Warburg effect in cancer and stromal cells to tumor-related autophagy may be of relevance for further development of experimental therapeutics as well as for cancer prevention.

## 1. Introduction

Autophagy is an evolutionarily conserved and highly regulated lysosomal pathway involved in the degradation of macromolecules and cytoplasmic organelles [1–3]. Autophagy is a crucial contributor to maintain cellular homeostasis. The quality control of mitochondria structure and function, for instance, is important in the maintenance of cell energy and this process seems to involve autophagy.

By 1920, Otto Warburg hypothesized that tumor cells mainly generate energy by nonoxidative breakdown of glucose, making cancer growth feasible. This phenomenon is known as “Warburg effect.” This cellular event relies on mitochondrial dysfunction, characterized by respiratory impairment, resulting in a switch to glycolysis.

Originally, the Warburg effect was thought to occur only in cancer cells. Nevertheless, in 2008, Vincent et al. demonstrated that human skin keloid fibroblasts display similar bioenergetic changes as cancer cells in generating ATP mainly from glycolysis. The hypoxic microenvironment is a common fact in solid tumors and keloids, which may be the explanation for this thermodynamic phenomenon [4]. In line with these findings, Pavlides and col suggested, in 2009, a novel hypothesis for understanding the Warburg effect in tumors [5]: they proposed that epithelial cancer cells induce the Warburg effect in neighboring stromal fibroblasts.

A clear association among mitochondrial function, Warburg effect, the reverse Warburg effect, and autophagy can be established. The objective of this review is to discuss the autophagy process, its regulation, the selective pathways, and its role in cancer cell metabolism. We define Warburg effect and the “reverse” hypothesis and we discuss the potential value of modulating autophagy. The relevance of these interactions on cancer cell biology will be also discussed, as well as their potential impact on disease prevention and treatment.

## 2. Autophagy and Cancer

Autophagy is a highly regulated-cellular pathway for degrading long-lived proteins and is the only known pathway for clearing cytoplasmic organelles. This process is involved in the turnover of long-lived proteins and other cellular macromolecules, and, when normal, it might play a protective role in development, aging, cell death, and defense against intracellular pathogens [6, 7]. Autophagy has been associated with a variety of pathological processes such as degenerative diseases (diabetes, neurodegenerative processes, etc.) and carcinogenesis, with highlights of biomedical relevance [8, 9].

Autophagy consists of several sequential steps: induction, autophagosome formation, autophagosome-lysosome fusion, and degradation. Three major types of autophagy exist in eukaryotes: (1) chaperone mediated autophagy (CMA), (2) microautophagy, and (3) macroautophagy, hereafter referred to as autophagy [10]. CMA allows the direct lysosomal import of unfolded, soluble proteins that contain a particular pentapeptide motif. In microautophagy, cytoplasmic material is directly engulfed into the lysosome at the surface of the lysosome by membrane rearrangement. Finally, autophagy involves the sequestration of cytoplasm into a double-membrane cytosolic vesicle, referred to as an autophagosome that subsequently fuses with a lysosome to form an autolysosome for the degradation by lysosomal hydrolases [11].

Autophagy is characterized by sequestration of bulk cytoplasm and organelles in double-membrane vesicles called autophagosomes, which eventually acquire lysosomal-like features [11, 12].

Autophagy is mediated by a set of evolutionarily conserved gene products (termed the ATG proteins) originally discovered in yeast [13]. In mammalian cells, BECN1 [2, 14–16] promotes autophagosome formation when it functions as part of a complex with the class III phosphatidylinositol 3-kinase (PI3K) mediating the localization of other autophagic proteins to the autophagosomal membrane [17]. However, despite the advances in understanding autophagy, autophagosome formation in mammalian cells is a complex process, and neither the molecular mechanisms nor all the implicated genes involved in its formation are fully elucidated.

More than 30 highly conserved genes that are involved in autophagy have been identified so far [18]. A core molecular machinery has been defined and is composed of four subgroups: first, the ATG1/unc-51-like kinase (ULK) complex; second, the class III phosphatidylinositol 3 kinase (PtdIns3K)/Vps34 complex I; third, two ubiquitin-like protein (ATG12 and ATG8 (LC3) conjugation systems; and four, two transmembrane proteins, ATG9/mATG9 (and associated proteins involved in its movement such as ATG18/WIPI-1) and VMP1 (whose expression triggers autophagy) [19–21]. Basal autophagy in unstressed cells is kept down by the action of the mammalian target of rapamycin complex 1 (mTORC1). Key upstream regulators of mTORC1 include the class I phosphoinositide 3-kinase (PI3K)-Akt pathway, which keeps mTORC1 active in cells with sufficient growth factors, and the AMP-activated protein kinase (AMPK) pathway that inhibits mTORC1 upon starvation and calcium signals [22, 23].

Autophagy is strongly induced in many types of cultured cells under stress conditions. These stress conditions include amino acid starvation. The effects of individual amino acids differ in their abilities to regulate autophagy. It has been suggested that amino acid starvation is followed by an activation of serine/threonine kinase mammalian target of rapamycin (mTOR) and the subsequent regulation of the class III PI3K. The mTOR is involved in the control of multiple cell processes in response to changes in nutrient conditions [24]. Particularly, mTOR complex 1 (mTORC1) requires Rag GTPase, Rheb, and Vps34 for its activation and the corresponding inhibition of autophagy in response to amino acids [25, 26]. AMP activated protein kinase (AMPK) senses energy levels and constitutes a key factor for cellular energy homeostasis. When energy levels are low, AMPK is activated. The activated AMPK then inactivates mTORC1 through TSC1/TSC2 and Rheb protein [27]. This inactivation of mTORC1 is an essential step for the induction of autophagy and plays a central role in autophagy. In addition to amino acid signaling, it has also been reported that other factors can regulate autophagy, such as hormones, growth factors, and many other factors, including bcl-2 [28], reactive oxygen species (ROS) [29], calcium [30], BNIP3 [31], p19ARF [32], DRAM [33], calpain [34], TRAIL [35], FADD [36], and myo-inositol-1,4,5-triphosphate (IP3) [37]. But it is important to point out that not all autophagy signals are transduced through mTOR signaling. A recent study showed that small-molecule enhancers of the cytostatic effects of rapamycin (called SMERs) induce autophagy independently of mTOR [38].

Depending on nutrient conditions, the activities of the ULK1 kinase complex can be regulated by mTOR. Under growing and high-nutrient conditions, the active mTORC1 interacts with the ULK1 kinase complex (ULK1-mATG13-FIP200-ATG101) and phosphorylates ULK1 and mATG13 and therefore inhibits the membrane targeting of the ULK1 kinase complex. On the other hand, during starvation condition, the inactivated mTORC1 dissociates from the ULK1 kinase complex. This dissociation results in the ULK1 kinase complex free to phosphorylate components, such as mATG13 and FIP200, in the ULK1 kinase complex, leading to autophagy induction [39].

The vacuole membrane protein 1 (VMP1), a pancreatitis-associated protein, is a transmembrane protein with no known homologues in yeast. Its expression induces autophagosome formation, even under nutrient-replete conditions while remaining an integrated autophagosomal membrane protein in mammalian cells [40]. Hyperstimulation of Gq-coupled CCK receptor in pancreatic acinar cells during acute pancreatitis [41] and mutated KRas in pancreatic cancer cells [42] induce VMP1 expression. In addition, VMP1 interacts with Beclin 1/ATG6 through its hydrophilic C-terminal region (VMP1-ATG domain), which is necessary for early steps of autophagosome formation [40, 43]. Besides, EPG-3/VMP1 is one of three essential autophagy genes conserved from worms to mammals. EPG-3/VMP1 regulates early steps of the autophagic pathway in* Caenorhabditis elegans* [44]. VMP1 along with ULK1 and ATG14 localizes in the endoplasmic reticulum-associated autophagosome formation sites in a PI3K activity-independent manner, confirming the key role of VMP1 in the formation of autophagosomes [19]. Interestingly, an accumulation of huge ubiquitin-positive protein aggregates containing the autophagy marker ATG8/LC3 was seen and p62 homolog [45] in* Dictyostelium* cells lacking Vmp1 gene showed. Moreover, the knockdown of VMP1 expression abolishes starvation and rapamycin-induced autophagosome formation [40]. It also abolishes autophagy induced by hyperstimulation of Gq-coupled CCK receptor in pancreatic acinar cells [41] or by chemotherapy in pancreatic tumor cells [46]. Furthermore, VMP1 is the only human disease-inducible ATG-protein described so far.

It has been shown that both downregulated and excessive autophagy have been implicated in the pathogenesis of diverse diseases. These diseases include a certain type of neuronal degeneration, diabetes and its complications, and cancer [47]. Autophagy has also been implicated in cell death called autophagic or type II programmed cell death, which was originally described on the basis of morphological studies detecting autophagic vesicles during tissue involution [48].

In general, cancer cells tend to undergo less autophagy than their normal counterparts, at least for some tumors [49, 50]. There is a monoallelic deletion of beclin1 autophagy gene in 40–74% of cases of human sporadic breast, ovarian, and prostate cancer [50]. Heterozygous disruption of beclin1 increases the frequency of spontaneous malignancies and accelerates the development of virus-induced premalignant lesions [50]. This suggests that defective regulation of autophagy promotes tumor genesis. It has been proposed that autophagy can suppress carcinogenesis by a cell-autonomous mechanism that involves the protection of genome integrity and stability and a nonautonomous mechanism that involves the suppression of inflammation and necrosis. On the other hand, autophagy may support the survival of rapidly growing cancer cells that have outgrown their vascular supply and are exposed to a hostile environment with an inadequate oxygen supply or metabolic stress. In contrast, excessive levels of autophagy promote cell death [51]. Accordingly, it has been proposed that autophagy can play an important role both in tumor progression and in promotion of cancer cell death [52]. For instance, in pancreatic ductal adenocarcinoma (PDAC), the cellular response to ROS initiates a survival or cell death pathway dependent on the severity of the oxidative damage [53]. ROS such as H_2_O_2_ can induce autophagy. The deregulation of the AGER ligand HMGB1 is expressed in many cancer cells including pancreatic cancer cells. ROS can increase the release of HMGB1 from necrotic cells and then activates Beclin-1-dependent autophagy by binding to AGER in pancreatic cancer cells [53, 54]. In addition, ROS can promote cytosolic translocation of HMGB1 to bind to Beclin-1 and then enhance autophagy [55]. Recent studies have demonstrated that oxidative stress increases the activity of NF-*κ*B which upregulates the expression of AGER in pancreatic cancer [56]. This in turn promotes autophagy flux by upregulation of LC3-II levels and protects pancreatic cancer cells from oxidative injury. On the other hand, ascorbate leads to cell death in PDAC through a unique ROS-mediated caspase-independent autophagy pathway [57], and gemcitabine and cannabinoids combination induces ROS-mediated autophagic cell death in pancreatic tumor cells [58].

There are suggestions that autophagy may be a cancer cell survival response to tumor-associated hypoxia. Tumor hypoxia has been used as a marker of poor prognosis [59]; in any case, how cancer cells become more malignant or survive with an extremely poor blood supply is poorly understood. When cancer cells are exposed to hypoxia, anaerobic glycolysis increases and provides energy for cell survival, but as the glucose supply is also insufficient due to the poor blood supply, there must be an alternative metabolic pathway that provides energy when both oxygen and glucose are depleted [60, 61]. In pancreatic cancer, there have been reports of hypoxia increasing the malignant potential of the tumor [59]. Proliferating cancer cells require more nutrients than surrounding noncancerous cells do. Nutrition of these proliferating cancer cells is supplied via functionally structurally immature neovessels. Autophagy may react to the cancer microenvironment to favor the survival of rapidly growing cancer cells. This is because autophagy-specific genes promote the survival of normal cells during nutrient starvation in all eukaryotic organisms. LC3 expression has been seen in surgically resected pancreatic cancer tissue that shows activated autophagy in the peripheral area, which included the invasive border and concomitantly exhibits enhanced expression of carbonic anhydrase [62]. This suggests that autophagy may promote cell viability in hypovascularized cancer tissue.

Another proposal is that autophagy is a survival cancer cell response to tumor-associated inflammation [63]. The promotion of carcinogenesis and resistance to therapy are two results of cancer-associated inflammation. Several phenotypic alterations observed in cancer cells are a result of inflammatory signals found within the tumor microenvironment [63]. The receptor for advanced glycation end products (RAGE) is an induced inflammatory receptor. It is constitutively expressed on many murine and human epithelial tumor cell lines [64, 65]. Murine and human pancreatic adenocarcinoma tumors have shown the highest levels of RAGE expression. Genotoxic and/or metabolic stress lead to modest but reproducible increases in overall expression of RAGE on epithelial cell lines. There is a direct correlation between RAGE expression and the ability of both murine and human pancreatic tumor cell lines to survive cytotoxic aggression. Targeted knockdown of RAGE significantly increases cell death, whereas forced overexpression promotes survival. It was recently reported that the enhanced sensitivity to cell death in the setting of RAGE knockdown is associated with increased apoptosis and decreased autophagy. In contrast, overexpression of RAGE is associated with an increased autophagy, but diminished apoptosis and enhances cancer cell viability. Knockdown of RAGE enhances mTOR phosphorylation in response to chemotherapy; therefore, there is a prevention of an induction of a survival response. Inhibition of autophagy by means of silencing beclin1 expression in pancreatic cancer cells enhances apoptosis and cell death [66]. These findings suggest that RAGE expression in cancer cells has a role in tumor cell response to environmentally induced stress through the enhancement of autophagy. However, increased sensitivity to chemotherapeutic agents in RAGE-knockdown pancreatic cancer cells is dependent on ATG5 expression but independent of BECN1 expression [66]. These last findings suggested that the role of autophagy in the resistance to microenvironment insult or in the sensitivity to chemotherapeutic agent is the result of complex molecular pathways in the tumor cell.

On the other hand, inhibition of autophagy has been suggested as a tumor cell response to prolonged hypoxic conditions. Pancreatic cancer cell response to prolonged hypoxia may consist in inhibition of autophagic cell death. A member of the basic helix-loop-helix family of transcriptional regulators [67], the short isoform of single-minded 2 (SIM2s), is upregulated in pancreatic cancer. The procell death gene BNIP3 has been identified by microarray studies as a target of SIM2s repression. Prolonged hypoxia induces cell death via an autophagic pathway involving the HIF1alfa-mediated upregulation of BNIP3 [31, 68]. There is an association between the deregulation of both SIM2s and BNIP3 with poor prognostic outcomes [69]. Decreased BNIP3 levels and poor prognosis correlate with elevated SIM2s expression in pancreatic cancer. The loss of BNIP3, either by hypermethylation or by transcriptional repression, correlates with inhibition of cell death [70, 71]. On the contrary, upregulation of BNIP3 sensitizes pancreatic carcinoma cells to hypoxia-induced cell death [72]. SIM2s expression, concomitant with its repression of BNIP3, enhances tumor cell survival under prolonged hypoxic conditions. Recent data linked the increased SIM2s expression with enhanced cell survival during hypoxia-stress associated with BNIP3 repression and the attenuation of hypoxia-induced autophagic processes. Therefore, inhibition of autophagic cell death by BNIP3 repression enhances tumor cell survival under prolonged hypoxic conditions.

In some cancer cells a relation between a decreased autophagy and malignant stages of the disease has been found. Cancer cells present a general tendency to undergo less autophagy than their normal counterparts; this supports the idea that defective autophagic cell death plays an important role in the tumor progression process. Pancreatic adenocarcinoma cells have lower autophagic capacity than premalignant cells. This has been proved by studies of carcinogen-induced pancreatic cancer in animal models [73]. The WIPI protein family, which includes ATG18, the WIPI-1 homolog in* S. cerevisiae*, was genetically identified as a gene contributing to autophagy [73]. Human WIPI-1a, a member of a highly conserved WD-repeats protein family, is linked to starvation-induced autophagic processes in the mammalian system. The deprivation of amino acids triggers an accumulation of endogenous hWIPI-1 protein. They are contained in large vesicular and cup-shaped structures where colocalize with LC3. The starvation-induced hWIPI-1 formation is blocked by wortmannin, a principal inhibitor of PI-3 kinase-induced autophagosome formation [74]. An interesting fact is that WIPI proteins are linked pathologically to cellular transformation. This is because all human WIPI genes are reported aberrantly expressed in a variety of matched human cancer samples. Strikingly, hWIPI-2 and hWIPI-4 mRNA expression is substantially decreased in 70% of matched kidney (10 patients) and 100% of pancreatic (seven patients) tumor samples. Most of these samples were derived from tumors in an advanced stage, such as pancreatic adenocarcinomas stages I–IV. Therefore, cancer-associated downregulation of hWIPI-2 and hWIPI-4 supports the possibility that decreased autophagic activity is necessary for the malignant stages of pancreatic cancer.

## 3. Warburg Effect and Cancer Cell Biology

Otto Warburg and colleagues performed studies measuring lactate production and oxygen consumption on liver rat carcinoma tissue and were able to propose that cancer cells display some very relevant differences when compared with normal tissues with regard to their glucose metabolism; glycolysis is favored despite oxygen availability. The hypothesis of Warburg was that cancer growth is caused by the fact that tumor cells mainly generate energy (in the form of ATP) by nonoxidative breakdown of glucose. This view contrasts with the observation that normal cells produce ATP during oxidative phosphorylation obtaining “fuel” through the oxidative breakdown of glucose [75].

Glycolysis under anaerobic condition produces 2ATP per molecule of glucose. This yield of ATP is much lower than the production of ATP by means of a complete oxidation of glucose to CO_2_ under aerobic conditions (30 or 32 ATP per molecule of glucose) [76]. In other words, about a 15 times higher amount of glucose is consumed anaerobically when compared to the aerobic pathway to yield the same amount of ATP. As consequence, glucose uptake takes place about ten times faster in most solid tumors than in normal tissues [77]. Commonly, cancer cells depend on anaerobic glycolysis for their ATP production due to their exposure to a limited O_2_ supply (hypoxia).

The “Warburg effect” was the denomination given to this phenomenon of preferred aerobic glycolysis, which results in an increased lactate production even in presence of adequate pO_2_. It was suggested that this cellular behavior relies on mitochondrial dysfunction, characterized by respiratory impairment, resulting into a switch to glycolysis. It was also suggested that the high glycolytic rate might also result from a decreased mitochondrial mass in tumor cells [78].

This effect, first described in cancer tissues, was further identified in many other rapidly dividing normal cells [79]. Several mechanisms have been proposed to explain the Warburg effect in cancer tissues and they may be involved in transcriptional and posttranslational related metabolic changes.

A reduced expression of the tumor suppressor protein p53 in cancer cells might be linked to the Warburg effect. P53 is known to reduce the glycolysis rate by increasing the activity of a fructose-2,6-bisphosphatase. This mechanism is also involved in the regulatory pathways of apoptosis [80, 81]. In addition, this mechanism seems to increase the oxidative phosphorylation process. Other transcription regulators, such as the alpha estrogen-related receptor (of potential relevance in breast cancer) might be linked to the Warburg effect; in the same way, an increased expression of oncogenes like MYC also seems to be associated with an increased glycolytic rate and might be involved in the pathophysiology of the metabolic modifications found in tumors [82]. Besides, glycolytic enzymes and glucose transmembrane transport are activated by MYC overexpression.

As mentioned before, the posttranslational regulation of the Warburg effect was also under scrutiny. As a relevant example, the activation of the PI3K/AKT downstream derives into an increased glucose influx and the phosphorylation of some enzymes like hexokinase and phosphofructokinase-2 with an upregulation of the glycolytic pathway [80]. Several posttranslational modifications of the M2 isoform pyruvate kinase result in a change in its activity, modulating the glycolytic pathway in several tissues. The K305 acetylation of this M2 isoform reduces its enzymatic activity and increases the enzyme degradation via chaperone-mediated autophagy [80]. The posttranscriptional modification of the M2 isoform of the pyruvate kinase has been shown to influence glycolysis at various models and experimental conditions, by oxidation, acetylation, phosphorylation, and so forth. A recent link was described among tumor overexpression of endogenous microRNA (miRNA), metabolic regulation of cancer cells, and the “Warburg effect” [80]. Even when attractive, the biological impact of this association remains to be clarified.

## 4. The Reverse Warburg Effect in Cancer: Pharmacological Modulation of Warburg and Reverse Warburg Effects

As stated before, it was thought that the Warburg effect only occurred in cancer cells. Recently, it has been shown that human skin keloid fibroblasts were able to generate energy (ATP) mainly from glycolysis; this phenomenon was explained through the existence of similar hypoxic microenvironments in tumors and keloids [4, 5]. This observation led to suggest a new hypothesis where epithelial cancer cells are able to induce the Warburg effect in stromal fibroblasts. This process was termed “reverse Warburg effect” and it is based in studies performed in cocultures systems (e.g., stromal fibroblasts and human breast cancer cells) [4]. This hypothesis is consistent with the original view and is important to point out that in this situation the Warburg effect is not occurring in cancer cells but in the stroma. For a clearer understanding, the reverse Warburg effect can be explained as occurring in two steps.

As a first step, cancer-associated fibroblasts undergo myofibroblastic differentiation and secrete lactate and pyruvate through the glycolytic pathway. As stated before, this process is induced in cancer cells by a mechanism involving oxidative stress in association with loss of Caveolin-1, mitophagy, and/or mitochondrial dysfunction and increased production of NO [83].

Following these changes, epithelial cancer cells take up the energy-rich metabolites, which in turn enter in the tricarboxylic acid (TCA) pathway. This leads to production of ATP by oxidative phosphorylation. The mitochondrial mass of these cells expands to satisfy the increased metabolic demand. In addition, antioxidant enzymes are upregulated in order to cope with the oxidative stress generated and increase tumor aggressive behavior [84].

It is conceivable that different variants of similar types of cancer may differ with regard to their metabolic behavior. Breast cancer seems to be heterogeneous in its metabolic status, and therefore it can be classified into various metabolic phenotypes. “Warburg” and mixed variants had been identified, closely associated with the triple negative breast cancer phenotype, whereas the reverse Warburg and null types were frequently identified within the luminal type of breast tumors, suggesting a correlation between metabolic phenotype and the biology of breast cancer [85].

The Warburg effect might be modified and reversed by some pharmacological interventions. Even though several mechanisms for such actions were reported, in general, the clinical relevance of these findings is still on the way of being clarified.

One of the most studied agents in this area is a well-known antidiabetic agent, metformin. This drug has been proposed as a potential multifaceted agent for cancer prevention. Metformin acts as an indirect activator of AMPK and is able to reduce mitochondrial complex I activity. These have been proposed as mechanisms for reducing hepatic glucose output in patients with type 2 diabetes. Metformin treatment was associated with an increased cell death in P53-deficient cancer cells. In normal cells, there is an increase in glycolysis rates as an alternative ATP-producing mechanism that follows metformin treatment. In fact, one very rare but still possible adverse effect of metformin is lactic acidosis. It seems that P53-deficient cells experience problems in switching their metabolic pattern. This is followed by an enhanced cell death rate. Metformin diminishes ROS generation at mitochondria [86]. This is mainly achieved by reducing the activity of the respiratory chain complex I. Acknowledging this is important because the role of ROS in tumorigenesis and in cancer growth has been widely recognized. Metformin exhibits a mild to moderate antiangiogenic effect; this is an effect that it shares with thrombospondin and endostatin. This effect on angiogenesis may be on the basis of its potential actions on cancer cells and/or its stroma [86].

In addition, as mentioned before, metformin activates the ATM/LKB1/AMPK axis. A very well-characterized tumor suppressor in the pathophysiology of melanoma and pancreatic and lung cancer, the tumor suppressor LKB1, might participate at the mechanism of action of metformin. It is thought that part of the preventive effects of metformin might be mediated by this suppressing factor. Metformin may inhibit the mTOR pathway by activating AMPK; this effect has been proposed as an explanation for the potential antineoplastic effects of metformin in breast and renal tumors [87]. Metformin's effects on the Warburg effect may be explained by many of the mentioned mechanisms. This drug has been suggested to reduce glycolysis and to increase mitochondrial respiration in tumors, and both effects have been associated with growth arrest [87]. It has been proposed that pyruvate kinase expression in fibroblast of tumoral stroma is linked to cancer growth. Cancer cells produce ROS that promote oxidative stress in fibroblasts. This results in the activation of HIF1 and NF-*κ*B. NF-*κ*B increases proinflammatory cytokines and HIF1 alpha promotes autophagy and anaerobic glycolysis. Pyruvate kinase activity results in an increase in ketones and lactate, and these nutrients are transferred to cancer cells where they are used for mitochondrial oxidative metabolism. As it has been said before, metformin reduces the mitochondrial chain activity by inhibiting complex I activity. In this manner, metformin may alter some of the mechanisms involved into the reverse Warburg effect [88]. It may also affect cell reprogramming by modifying the lipogenic enzymes acetyl-Co A carboxylase and fatty acid synthase [89]. These changes may also affect the metabolic behavior of both stroma and tumor cells. As mentioned before, the clinical impact of these modifications is still uncertain.

There are other drugs that exhibit potential for the modification of Warburg effect and authophagy rates. Mild autophagy induction by hypoxia or starvation seems to protect the cells, but rapamycin or sulforaphane leads to its elimination [90]. In contrast, an excessive autophagy rate may induce cell death. Elimination of highly aggressive pancreatic adenocarcinoma cells can be achieved by inhibition of autophagy by monensin or 3-methyladenine [90]. This is possible because these drugs may totally block continuous recycling of cellular components necessary for new synthesis and survival. This information suggests that both inhibitors and activators of autophagy may have utility in the treatment of patients with pancreatic ductal adenocarcinoma, since strong overactivation as well as strong inhibition of autophagy induces death in highly aggressive adenocarcinoma cells and sensitizes them to hypoxia-starvation [90]. Both autophagy activating (e.g., rapamycin—derivates sirolimus and temsirolimus or sulforaphane-a naturally occurring dietary substance enriched in broccoli) or inhibiting drugs (e.g., antibiotic monensin, antimalarial drug chloroquine) are available and generally tolerated well by patients.

Autophagy may be necessary for the maintenance of the tumor in advanced cancer. This is why multiple clinical trials are on their way to test this as a therapeutic approach in human patients using hydroxychloroquine (HCQ) [91, 92].

Autophagy can be affected in different manner and several ways by standard cancer chemotherapies. Gemcitabine monotherapy or its combination with other agents has become the standard chemotherapy for the treatment of advanced pancreatic cancer. Gemcitabine is a relatively effective chemotherapeutic agent acting by competition with dCTP for the incorporation into DNA causing chain termination. On the other hand, gemcitabine serves as an inhibitory alternative substrate for ribonucleotide reductase and leads to a reduction of deoxynucleotide pools [93, 94]. This molecule inhibits cells that are insensitive to classic anticancer drugs, including other nucleoside analogs with similar structures. It has been recently suggested that gemcitabine also induces autophagy in pancreatic cancer cells [46] even though gemcitabine seems to exert its toxicity at least in part by activation of apoptosis [93]. It has been proposed that the early induction of autophagy with gemcitabine may be mediated by an increased expression of VMP1 [46]. Capecitabine, which is a pyrimidine analog, induces apoptosis in several cancer lines and shows a modest efficacy in locally advanced pancreatic ductal adenocarcinoma when associated with limited field radiotherapy [95]. It has been proposed that capecitabine modulates autophagy by displaying a Src kinase modulatory effect [96], but the results on this area are still contradictory. Irinotecan is a topoisomerase I inhibitor which prevents DNA from unwinding. In a phase III trial, the combination of 5-fluouracil, leucovorin, oxaliplatin, and irinotecan resulted in better responses, progression-free survival, and overall survival when compared with the standard single drug therapy with gemcitabine for metastatic pancreatic ductal adenocarcinoma [97]. In small-cell prostatic carcinoma, irinotecan promoted an increase in autophagy of treated tumors as indicated by an increase in LC3B expression [98, 99]. Nevertheless, authors of this research state that the role of autophagy is complex. This can be said because there is evidence that autophagy supports both promotion and suppression of cancer growth. In general, as mentioned before, a considerable amount of caution should be exercised for the interpretation of the consequences of cancer chemotherapy on autophagy. Other chemotherapeutic agents like the glycoside oleandrin, some platinum compounds, the multikinase inhibitor sorafenib, and some histone-deacetylase inhibitors have demonstrated effects on the autophagy rate in pancreatic carcinoma cell lines [98, 99]. As proposed, autophagy may be involved in carcinogenesis, tumor progression, and dissemination and may be associated at least in part with the actions of some chemotherapy for pancreatic ductal adenocarcinoma as well. All these modifications may alter Warburg and reverse Warburg effects, but it is important to remember that the real contribution of these metabolic changes to tumor cell survival and clinical prognosis remains unclear.

## 5. Conclusions

**Figure 1:**
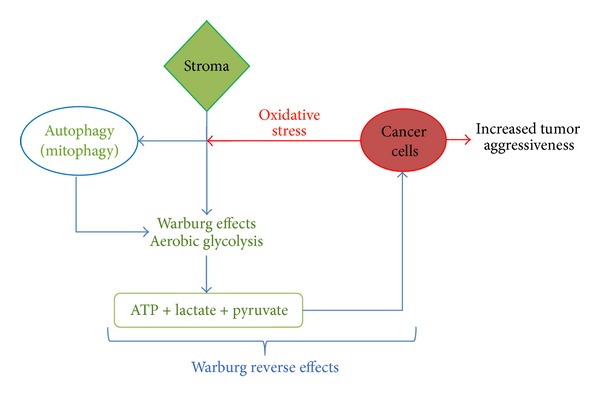
Autophagy, Warburg, and Warburg reverse effects in human cancer. The link between abnormalities in autophagy and the Warburg and the reverse Warburg effects seems to be critical to understand several tumor adaptive behaviors.

Autophagy regulation involves a set of key processes needed for a normal cell survival and turnover. The association between abnormal or defective autophagy and cancer development has been strongly suggested by several authors. This association is currently under an intense scrutiny aimed to contribute to a better understanding of the tumor cell biology.  Figure 1 summarizes the link between abnormalities in autophagy and the Warburg and the reverse Warburg effects, critical to understand several tumor adaptive behaviors. A better knowledge of these metabolic interactions may be of importance in the development of new therapeutic agents in oncology, as well as for the development of more efficient preventive strategies for some cancer phenotypes.
